# Notes and News

**Published:** 1901-10-12

**Authors:** 


### Notes and News.

Dr. Norman Moore will deliver the Haweian Oration, before the Royal College of Physicians, on October 18th, at four o'clock.

The Glasgow Royal Asylum will much miss the services of Dr. Yellowlees, who has for 27 years held the post of physician superintendent. Dr. Yellowlees has been obliged to resign owing to an affection of the eyes.

Lectures and clinical demonstrations will be delivered at the Hospital for Sick Children, Great Ormond Street, every Thursday afternoon at four o'clock until December 19th. The lecture next week will be given by Dr. Still on diarrhœa in infants.

The honorary secretaries of the Prince of Wales's Hospital Fund for London have received at the Bank of England the sum of 100 guineas from the King, who has been pleased to continue the annual subscription which His Majesty—as Prince of Wales—has hitherto contributed to this fund.

The winter session of the West London Post-graduate Course will commence on Monday, October 14th, when an address will be delivered by Sir William MacCormac, Bart., K.C.V.O. The demonstrations at the National Hospital for the Paralysed and Epileptic, Queen's Square, which take place at 2.30 p.m., have already recommenced. Many other special hospitals also have arranged courses of lectures so that many opportunities for post-graduate study are now open to practitioners in London.

The India Plague Commission continues to receive reports from different districts on the result of inoculation with Haffkine's plague serum. A uniform statistical conclusion has not been arrived at, but nevertheless the conclusion may be drawn from the arguments and observations within the reports, that inoculation has shown sufficiently good results to warrant unrelaxed perseverance in popularising it throughout India. The native population are far less averse to it than to disinfecting or segregation methods of exterminating the disease, even though they have to pay for treatment themselves.

Mr. H. G. Durham, M.B., F.R.C.S., and Mr. P. Manson, M.R.C.S., L.R.C.P., are going to undertake the inquiry into beri-beri at Christmas Island proposed by and largely endowed by the Phosphate Company.

A new workhouse infirmary has been erected at Retford. It contains two acute wards for male and female patients, with 56 beds. Above these wards are two more wards and day rooms, with balconies for infirm inmates. There is also a maternity ward. Dispensary, offices, and nurses' quarters complete the building, which cost £4,000.

At the Lytham Cottage Hospital a new ward has been added. It is called the Fisher Memorial Ward, as it is erected to the memory of Dr. Luke Fisher, who gave his services to the hospital as honorary medical superintendent and secretary for nearly 30 years. He was the first chairman of the hospital, and was instrumental in its establishment.

It is reported that Dr. Caldas does not regard the test of his yellow fever serum as satisfactory, and repudiates the theory that patients made immune by it died from the fever transmitted by a mosquito. He does not think he has been fairly treated by the United States Government. He has been recalled by the Brazilian Government to conduct experiments in Rio de Janeiro.

How to allay typhoid fever at Belfast is a riddle still far from solution, judging from the results of the recent meeting of the Borough Council. There is no real diminution in its prevalence, though the authorities have been stiffening regulations, and making numerous inquiries and receiving reports. The Local Government Board is to be asked to sanction a loan of £50,000 to build an infectious diseases hospital.

All physicians and surgeons to the London Hospital retire after 20 years' service from the date of their attaining their full rank. We understand that Dr. Stephen Mackenzie, the senior physician, was appointed full physician at this hospital on September 14th, 1886, and assistant physician, March 17th, 1874, and that Mr. Waren Tay, the senior surgeon, was appointed full surgeon on June 27th, 1882, and assistant surgeon on April 14th, 1869.

The Order of the League of Mercy has, with the consent of the King, been conferred upon the following ladies and gentlemen:—Marquis of Camden, Earl of Mansfield, Viscountess Parker, Lady Pirbright, Lady Farquhar, the Hon. Mrs. Halford, Lady Maclean, Lady Faudel Phillips, Lady Treloar, Mr. R. C. Antrobus, Mr. A. H. Baker, Mrs. Nettleton Balme, Mrs. Boscawen, Mrs. Charles Davis, Mr. T. C. Dewey, Colonel C. A. Gorham, Colonel H. B. Hamilton, Mr. James Harper, M.D., Alderman John Harris, Mrs. R. L. Harrison, Mrs. Edwin Hughes, Mr. P. Cremieu Javal, Mr. T. B. Jobson, Mr. J. C. Marshall, Mrs. Buxton Morrish, Colonel Arther Allen Owen, Mr. H. J. Palmer, Miss Clara Seligman, Captain A. E. Speer, Mr. Alfred H. Tarleton, Miss Mary B. Thompson.

